# Quantra® Viscoelastic Testing in Pediatric Trauma: A Review of Emerging Evidence and Clinical Integration

**DOI:** 10.7759/cureus.99134

**Published:** 2025-12-13

**Authors:** Chris Deskins, Samantha Smith, Patrick C Bonasso, William S Jones, Ryan I Bower, Marcus Bowers, Zachary Kochy, Alice Seifarth, Erik Olness, Pavithra R Ellison

**Affiliations:** 1 Anesthesiology, West Virginia University, Morgantown, USA; 2 Pediatric Surgery, West Virginia University, Morgantown, USA; 3 Trauma, WVU Medicine, Morgantown, USA

**Keywords:** bleeding, injury, pediatric trauma, quantra® viscoelastic testing, trauma-induced coagulopathy

## Abstract

Early recognition and correction of trauma-induced coagulopathy (TIC) are critical determinants of survival in pediatric trauma. Traditional laboratory evaluations, such as prothrombin time (PT)/international normalised ratio (INR) and fibrinogen, provide a delayed and incomplete assessment of real-time clotting behavior. The Quantra® System (HemoSonics, Charlottesville, VA, USA), a type of viscoelastic hemostatic assay (VHAs), utilizes soundwave technology to provide a more rapid and comprehensive assessment of coagulation. This review summarizes current evidence regarding Quantra use in pediatric trauma, compares it with conventional viscoelastic tools (TEG® (Haemonetics, Boston, MA, USA) and ROTEM® (Werfen, Bedford, MA, USA)), and discusses emerging experience in perioperative monitoring and trauma resuscitation pathways. Though preliminary data suggests Quantra is well-correlated with conventional VHAs and offers rapid, simple, and standardized assessment in pediatric trauma resuscitation, large-scale pediatric validation studies are needed to define thresholds, optimize transfusion algorithms, and assess outcomes.

## Introduction and background

Trauma remains the leading cause of morbidity and mortality among children older than one year of age [1]. In one study of over 800 patients, early coagulopathy occurred in 37.9% with a fourfold increase in mortality when associated with traumatic brain injury (TBI) [2]. Traditional coagulation assays (prothrombin time (PT), activated partial thromboplastin time (aPPT), fibrinogen, platelets) require exposure to multiple reagents and machine counting by trained professionals and are limited by potentially long turnaround times (an hour or more) in a lab, poor correlation with ongoing bleeding, and user error [3]. These tests were developed to monitor chronic, isolated coagulopathies such as hemophilia, rather than as diagnostic tools to treat acute coagulopathy in real time [4,5]. In addition, traditional coagulation tests measure only the initial phase of hemostasis and thrombin generation, omitting critical information about clot strength, stability, and breakdown over time [5,6]. This lack of real-time data may misguide resuscitation efforts in an active pediatric trauma [7].

Viscoelastic hemostatic assays (VHAs) overcome many of these limitations by providing real-time dynamic assessment of clot initiation, strength, and lysis through mechanical or resonance-associated mechanisms under strictly standardized and regulated conditions. As a result, studies show reduced mortality with VHA-guided management compared to conventional coagulation assays [7,8]. The Quantra® Hemostasis Analyzer (HemoSonics, Charlottesville, VA, USA) utilizes a proprietary ultrasound-based technology that quantifies clot stiffness from a whole-blood sample by employing sonic estimation of elasticity via resonance (SEER) sonorheometry [9]. This technology provides a rapid (< than 15 minutes) and comprehensive model of the patient’s ability to progress through the coagulation cascade. Quantra ultimately enhances goal-directed resuscitative accuracy in dynamic and life-threatening pathology [9].

In adult trauma, Quantra® has been validated against ROTEM® (Werfen, Bedford, MA, USA) and TEG® (Haemonetics, Boston, MA, USA) and is now integrated into several massive transfusion protocols [6,9]. Unfortunately, pediatric-specific evidence remains limited. This review synthesizes current evidence and institutional experience, highlighting Quantra’s role in early detection of coagulopathy, targeted transfusion, and perioperative decision-making. Our authors intend to provide a narrative review summarizing emerging evidence and integration of Quantra into pediatric trauma workflows.

## Review

Principles of SEER sonorheometry 

Unlike optical or mechanical detection methods used in ROTEM® and TEG®, the Quantra system uses ultrasound pulses to quantify clot stiffness [9]. The analyzer measures how blood viscoelasticity changes during clotting, producing parameters such as clot time (CT, initiation of clot formation), clot stiffness (CS, overall mechanical integrity), platelet contribution to clot stiffness (PCS), fibrinogen contribution to clot stiffness (FCS), and clot stability to lysis (CSL, resistance to fibrinolysis). These parameters result within 10-15 minutes of sample collection. This is faster than traditional VHAs (20-30 minutes) and markedly faster than standard coagulation assays (45+ minutes) [9,10]. SEER has been previously investigated for use in cardiac surgery, major orthopedic surgery, liver transplant, and adult trauma, with results suggesting overall favorable use [11-13].

Clinical relevance in pediatric trauma 

Pediatric trauma patients can present with dilutional or consumptive coagulopathy following injury, blood loss, and resuscitation. Those patients presenting with coagulopathy have a 12% chance of mortality compared to 3% in non-coagulopathic children, and the chance of mortality increases to 31% if a pediatric trauma patient also experiences TBI [14]. The ability for rapid correction of coagulopathy may infer marked improvement in mortality for pediatric trauma patients with and without traumatic brain injury, though more research is required to analyze the direct mortality reduction [2,15]. Quantra offers rapid bedside quantification of coagulopathy, including functional fibrinogen and platelet contributions to allow targeted correction before clinical bleeding occurs [2,16]. At our institution, a trauma level II children’s hospital, we have clinically demonstrated the feasibility of obtaining Quantra results within 10 minutes of arrival, quickly guiding transfusion decisions alongside clinical judgment. For example, early FCS depression correlated with hypofibrinogenemia (Clauss assay, <150 mg/dL), supporting Quantra’s utility as a rapid fibrinogen screen. Importantly, Quantra’s adult reference ranges appear to be applicable to pediatric patients from one year of age and older [17].

Comparison with ROTEM and TEG 

Comparative analyses demonstrate a strong correlation between Quantra parameters and various ROTEM measures. In one study, Quantra’s CS correlated with ROTEM A10 (r ≈ 0.85-0.90), and Quantra’s FCS with FIBTEM A10 (r ≈ 0.82-0.88) [6,9]. Moore et al. observed shorter clot times and higher clot stiffness in Quantra compared to ROTEM and TEG [6]. This was thought likely due to the oscillatory forces and shear stresses experienced in ROTEM and TEG, causing depressed results [6]. Multiple studies show moderate-to-strong concordance of Quantra with TEG and ROTEM in detection of hyperfibrinolytic states [6,12,13,18]. SEER also appears to more accurately model fibrinolysis in the presence of antifibrinolytic therapy (i.e., tranexamic acid), which is frequently administered in early trauma encounters [6,19]. Additional advantages of Quantra include standardized cartridges to minimize operator variability, closed-system sampling which improves safety and ease-of-use, rapid turnaround, and a compact footprint allowing for use in operating rooms and trauma bays with limited space [9].

Perioperative and trauma workflow integration

SEER technology has utility in high-risk surgeries and trauma as the system uses an ultrasound-based technology that does not require moving parts or direct contact with the sample being measured while enabling a comprehensive set of internal quality control measures [12]. Quantra is increasingly used in pediatric cardiac, craniofacial, and neurosurgical procedures [20]. Its speed and automation support intraoperative hemostatic monitoring. Integration with perioperative blood management dashboards allows real-time feedback to anesthesia and surgical teams. Quantra’s rapid display of real-time results can allow for early detection and treatment of coagulopathy and has been shown to reduce transfusion needs [11,17]. For patients in the ICU and on ECMO, the benefits of Quantra can continue to be employed for monitoring and continued resuscitation following the initial stabilization effort [21]. 

Point-of-care viscoelastic data can also improve anticipated need and accuracy in massive transfusion protocols (MTPs). Adult MTP algorithms incorporating Quantra have reduced plasma and platelet overuse without increasing bleeding complications [6]. Goal-directed resuscitation guided by real-time data is crucial in pediatric patients as over-transfusion risks volume overload and alloimmunization [14]. Thresholds of FCS <1.0 hPa and PCS <10 hPa have been proposed for cryoprecipitate (fibrinogen) and platelet transfusion, respectively, though pediatric validation remains pending [22]. 

At our institution, Quantra integration into the pediatric trauma workflow is implemented as part of a quality improvement initiative focused on early recognition of coagulopathy and targeted transfusion, mirroring adult trauma center protocols but adapted for pediatric weight-based resuscitation [3,21]. Advantages for pediatric use include minimal sample volume (one 3ml sample vs multiple vials of 500ul - 3ml samples measuring individual assays), rapid turnaround, ease of training (cartridge-based system minimizes educational requirements), consistency across multiple settings (OR, ICU, ED), and electronic integration [10,21,23]. Our Level 2 trauma center sees about 200 trauma patients a year, of which approximately 20 patients have needed the Quantra test for blood transfusion guidance. 

Limitations and future directions 

Despite encouraging feasibility data, limitations include large-scale pediatric validation trials [20,24], variable transfusion thresholds (neonates and infants), limited data correlating metrics with clinical bleeding outcomes, and availability in resource-limited settings [9,20]. Most pediatric trauma recommendations are derived from the pediatric cardiac surgery populations [24]. Ongoing multi-center studies (e.g., PEdi-VHA Consortium, 2024-2026) aim to define normative pediatric Quantra profiles and outcome-based cutoffs [25]. 

The evolution of viscoelastic testing in pediatric trauma lies in algorithmic integration, combining Quantra metrics with prehospital triage guidelines (e.g., Pediatric Trauma Score, Shock Index Pediatric Adjusted) to guide transfusion and antifibrinolytic decisions [3,22]. Integration with artificial intelligence-driven dashboards could further improve prediction accuracy for bleeding risk and transfusion need. Institutional quality improvement projects using Quantra also serve as educational tools to improve interdisciplinary understanding of hemostasis, and reduce empiric transfusion [3,21]. 

## Conclusions

Quantra viscoelastic testing represents a promising advancement in point-of-care hemostasis management for pediatric trauma. Its simplicity, rapid turnaround, and strong correlation with established VHAs make it well-suited for integration into emergency and perioperative workflows. While early data are encouraging, further pediatric validation studies are essential to establish age-adjusted reference values and outcome-based transfusion thresholds. As part of a multidisciplinary trauma resuscitation strategy, Quantra has the potential to enhance precision transfusion, reduce blood product utilization, and improve outcomes in injured children. 
